# Antihyperglykämische Therapie bei Diabetes mellitus Typ 2 (Update 2026)

**DOI:** 10.1007/s00508-026-02716-w

**Published:** 2026-04-30

**Authors:** Martin Clodi, Heidemarie Abrahamian, Felix Aberer, Alexander Bräuer, Kadriye Aydinkoc-Tuzcu, Helmut Brath, Johanna Brix, Heinz Drexel, Peter Fasching, Astrid Feder, Lisa Frühwald, Maria Fritsch, Bernhard Föger, Claudia Francesconi, Elke Fröhlich-Reiterer, Jürgen Harreiter, Sabine Hofer, Florian Höllerl, Friedrich Hoppichler, Joakim Huber, Simone Huber, Susanne Kaser, Alexandra Kautzky-Willer, Antonia Kietaibl, Gerd Köhler, Monika Lechleitner, Michael Leutner, Bernhard Ludvik, Julia K. Mader, Verena Parzer, Bernhard Paulweber, Thomas Pieber, Rudolf Prager, Elisabeth Pucher, Birgit Rami-Merhar, Gersina Rega-Kaun, Michael Resl, Claudia Ress, Michaela Riedl, Michael Roden, Christoph H. Saely, Christian Schelkshorn, Guntram Schernthaner, Harald Sourij, Lars Stechemesser, Thomas Steinmaurer, Harald Stingl, Thomas Stulnig, Martin Tauschmann, Hermann Toplak, Gerlies Treiber, Alexander Vonbank, Thomas C. Wascher, Raimund Weitgasser, Yvonne Winhofer, Sandra Zlamal-Fortunat

**Affiliations:** 1 Abteilung für Innere Medizin, Konventhospital der Barmherzigen Brüder Linz, Klinisches Forschungsinstitut für kardiovaskuläre und metabole Erkrankungen JKU Linz, Linz, Österreich; 2 Endokrinologische Ordination, Wien, Österreich; 3 Universitätsklinik für Innere Medizin, Klinische Abteilung für Endokrinologie und Diabetologie, Medizinische Universität Graz, Graz, Österreich; 4 5. Medizinische Abteilung für Endokrinologie, Rheumatologie und Akutgeriatrie, Wiener Gesundheitsverbund, Klinik Ottakring, Wien, Österreich; 5 5. Medizinische Abteilung mit Endokrinologie, Rheumatologie und Akutgeriatrie, Klinik Ottakring, Wien, Österreich; 6 Sigmund Freud Privatuniversität Medizin, Wien, Österreich; 7 Diabetes- und Fettstoffwechselambulanz, Mein Gesundheitszentrum Favoriten, Wien, Österreich; 8 1. Medizinische Abteilung mit Diabetologie, Endokrinologie und Nephrologie, Klinik Landstraße, Wien, Österreich; 9 Vorarlberg Institute for Vascular Investigation and Treatment (VIVIT), Feldkirch, Österreich; 10 Universitätsklinik für Kinder- und Jugendheilkunde, Abteilung für Allgemeine Pädiatrie, Medizinische Universität Graz, Graz, Österreich; 11 Abteilung für Allgemein Innere Medizin, Rottal Inn Kliniken, Pfarrkirchen, Deutschland; 12 Rehabilitationszentrum Alland PV, Alland, Österreich; 13 Abteilung für Innere Medizin mit Palliative Care, Landesklinikum Scheibbs, Scheibbs, Österreich; 14 Abteilung für Kinder- und Jugendheilkunde Pädiatrie I, Endokrinologie, Medizinische Universität Innsbruck, Innsbruck, Österreich; 15 1. Medizinische Ambulanz für Fettstoffwechselstörungen, Wiener Gesundheitsverbund, Klinik Landstraße, Wien, Österreich; 16 Abteilung für Innere Medizin, Krankenhaus der Barmherzigen Brüder Salzburg, Salzburg, Österreich; 17 Diabeteszentrum am Wienerberg, 1. Medizinische Abteilung, Klinik Landstraße, Wien, Österreich; 18 1. Medizinische Abteilung für Endokrinologie, Diabetologie und Stoffwechsel, Klinik Landstraße, Wien, Österreich; 19 Universitätsklinik für Innere Medizin I, Medizinische Universität Innsbruck, Innsbruck, Österreich; 20 Klinische Abteilung für Endokrinologie und Stoffwechsel, Universitätsklinik für Innere Medizin III, Medizinische Universität Wien, Wien, Österreich; 21 5. Medizinische Abteilung für Endokrinologie, Rheumatologie und Akutgeriatrie, Klinik Ottakring, Wien, Österreich; 22 Rehabilitationszentrum Aflenz für Stoffwechselerkrankungen mit Schwerpunkt Diabetes mellitus und Adipositas, Aflenz, Österreich; 23 Avomed – Arbeitskreis für Vorsorgemedizin und Gesundheitsförderung in Tirol, Innsbruck, Österreich; 24 1. Medizinische Abteilung mit Diabetologie, Endokrinologie und Nephrologie und Karl Landsteiner Institut für Adipositas und Stoffwechselerkrankungen, Klinik Landstraße, Wien, Österreich; 25 Klinische Abteilung für Endokrinologie und Diabetologie, Universitätsklinik für Innere Medizin, Medizinische Universität Graz, Graz, Österreich; 26 Universitätsklinik für Innere Medizin 1, Landeskrankenhaus Salzburg – Universitätsklinikum, Paracelsus Medizinische Privatuniversität, Salzburg, Österreich; 27 Klinische Abteilung für Endokrinologie und Diabetologie, Medizinische Universität Graz, Graz, Österreich; 28 Ordinationszentrum an der Wiener Privatklinik, Wien, Österreich; 29 5. Medizinische Abteilung für Endokrinologie, Rheumatologie und Akutgeriatrie, Wilhelminenspital Wien, Wien, Österreich; 30 Universitätsklinik für Kinder- und Jugendheilkunde, Abteilung für Pulmonologie, Allergologie und Endokrinologie, Medizinische Universität Wien, Wien, Österreich; 31 Abteilung für Innere Medizin, Krankenhaus Barmherzige Brüder Linz, Klinisches Forschungsinstitut für kardiovaskuläre und metabole Erkrankungen JKU Linz, Linz, Österreich; 32 Abteilung für Innere Medizin I, Medizinische Universität Innsbruck, Innsbruck, Österreich; 33 Klinik für Endokrinologie und Diabetologie, Medizinische Fakultät und Universitätsklinikum, Heinrich-Heine-Universität, Düsseldorf, Deutschland; 34 Private Universität im Fürstentum Liechtenstein, Triesen, Liechtenstein; 35 1. Medizinische Abteilung, Landesklinikum Stockerau, Stockerau, Österreich; 36 1. Medizinische Abteilung mit Diabetologie, Endokrinologie und Department für Nephrologie, Krankenanstalt Rudolfstiftung, Wien, Österreich; 37 Klinische Abteilung für Endokrinologie und Diabetologie, Cardiometabolic Trials Unit, Medizinische Universität Graz, Graz, Österreich; 38 Universitätsklinik für Innere Medizin I mit Gastroenterologie-Hepatologie, Nephrologie, Diabetologie und Stoffwechselerkrankungen, Medizinische Universität Wien, Wien, Österreich; 39 Universitätsklinikum, Paracelsus Medizinische Privatuniversität, Salzburg, Österreich; 40 Abteilung für Innere Medizin, Landesklinikum Baden-Mödling, Baden, Österreich; 41 3. Medizinische Abteilung – Innere Medizin mit Stoffwechselerkrankungen und Nephrologie, Wiener Gesundheitsverbund, Klinik Hietzing, Wien, Österreich; 42 Universitätsklinik für Kinder- und Jugendheilkunde, Abteilung Pädiatrische Diabetologie, Medizinische Universität Wien, Wien, Österreich; 43 Universitätsklinik für Innere Medizin, Medizinische Universität Graz, Graz, Österreich; 44 Abteilung Innere Medizin I und Intensivmedizin, Krankenhaus Feldkirch, Feldkirch, Österreich; 45 1. Medizinische Abteilung, Mein Hanusch-Krankenhaus, Wien, Österreich; 46 Kompetenzzentrum Diabetes, Mavie Med Privatklinik Wehrle-Diakonissen, Salzburg, Österreich; 47 Abteilung für Innere Medizin und Gastroenterologie, Hepatologie, Endokrinologie, Rheumatologie und Nephrologie, Klinikum Klagenfurt, Klagenfurt, Österreich

**Keywords:** Diabetes mellitus Typ 2, Therapie, Blutzuckersenkung, Diabetes mellitus, type 2, Treatment, Glycemic control

## Abstract

Die Hyperglykämie ist wesentlich an der Entstehung der Folgeerkrankungen bei Menschen mit Diabetes mellitus Typ 2 beteiligt. Während Lebensstilmaßnahmen die Eckpfeiler jeder Diabetestherapie bleiben, benötigen die meisten Menschen mit Typ-2-Diabetes im Verlauf eine medikamentöse Therapie. Bei der Definition individueller Behandlungsziele stellen die Therapiesicherheit, die Effektivität sowie substanzspezifische, organprotektive Effekte der Therapie die wichtigsten Faktoren dar. Diese nationale Leitlinie fasst die Evidenz aus der aktuellen Datenlage für die klinische Praxis zusammen.

## Abstract (en)

Hyperglycemia is substantially involved in the occurrence of complications in people with type 2 diabetes mellitus. While lifestyle interventions remain the cornerstones of diabetes treatment, most people with type 2 diabetes will eventually require pharmacotherapy for improved glycemic management. The definition of individual treatment targets regarding optimal therapeutic efficacy and safety as well as organ-protective effects are the most important factors. These national guidelines summarize the most current evidence-based recommendations for the clinical practice.

## Einleitung

Bei Menschen mit Diabetes mellitus Typ 2 trägt die Hyperglykämie entscheidend zur Pathogenese vaskulärer Komplikationen sowie Organkomplikationen bei, ist Kofaktor bei der Entwicklung makrovaskulärer Erkrankungen und ist ursächlich für direkte zelluläre Schädigungen.

Das primäre Ziel einer antihyperglykämischen Therapie ist daher neben dem Vermeiden von akuten Komplikationen der Hyperglykämie die Prävention von Folgeerkrankungen. Symptomfreiheit und der Erhalt der Lebensqualität stellen weitere, wesentliche Therapieziele dar.

### Therapieziele

- Vermeiden von Folgeschäden
- Vermeiden von Akutkomplikationen
- Symptomfreiheit sowie Erhalt bzw. Wiederherstellung der Lebensqualität

Die Bedeutung von Prädiabetes als kardiovaskulärer Risikofaktor ist durch die Analyse großer Registerstudien zusehends in den Vordergrund gerückt. Demnach ist bereits ein HbA_1c_ im prädiabetischen Bereich signifikant mit einem höheren Risiko für kardiovaskuläre Ereignisse assoziiert. Selbst erhöhte HbA_1c_ Werte innerhalb des normoglykämischen Bereichs zeigen epidemiologisch ein erhöhtes, kardiovaskuläres Ereignisrisiko [1].

Unverändert stellt die Umsetzung lebensstilmodifizierender Maßnahmen mit den Zielen Gewichtsreduktion und Muskelaufbau/-erhalt die Grundlage der Therapie des Diabetes/Prädiabetes dar.

Bei Menschen mit Adipositas sollte die Gabe eines GLP-1-Agonisten bzw. GIP/GLP-1-Agonisten zur effektiven Vermeidung des Auftretens eines Prädiabetes bzw. Diabetes mellitus Typ 2 in Erwägung gezogen werden.

In der SELECT-Studie reduzierte Semaglutid bei Menschen mit Adipositas das Auftreten von Diabetes innerhalb eines Beobachtungszeitraums von 34 Monaten um 73 %, auch kardiovaskuläre Ereignisse konnten signifikant um 20 % reduziert werden [2]. Auch der duale GIP/GLP-1-Agonist Tirzepatid reduzierte in der SURMOUNT 1-Studie die Manifestation eines Diabetes mellitus Typ 2 innerhalb von 172 Wochen von 13,3 % auf 1,3 %, einer Risikoreduktion für das Auftreten eines Diabetes von 93 % entsprechend [3].

Für die antihyperglykämische Therapie gelten die unten angeführten Zielwerte. Als Mittel der ersten Wahl bei Menschen mit Diabetes mellitus Typ 2 ohne Komorbidität sollte Metformin eingesetzt werden.

Bei Patient:innen mit Komorbiditäten sind SGLT-2-Hemmer und GLP1-RA, GLPR1/GIP-RA die Mittel der ersten Wahl, Metformin sollte aber, so es keine Kontraindikation gibt, bestmöglich bereits als initiale Kombinationstherapie zur Anwendung kommen.

Bei Kontraindikationen oder Unverträglichkeiten eines der genannten Medikamente muss je nach individuellen Erfordernissen des Patienten ein anderes der verfügbaren Präparate angewandt werden.

Sollte bei einer Monotherapie mit einem Medikament der individuelle HbA_1c_-Zielwert innerhalb von 3 Monaten nicht erreicht werden, muss eine Therapiemodifikation durchgeführt werden.

Liegt der HbA_1c_-Wert 1,5–2,0 % über dem individuell festgelegten Zielwert, so wird generell eine Kombinationstherapie zur raschen Erreichung des HbA_1c_-Ziels empfohlen [4].

**Abb. 1:**
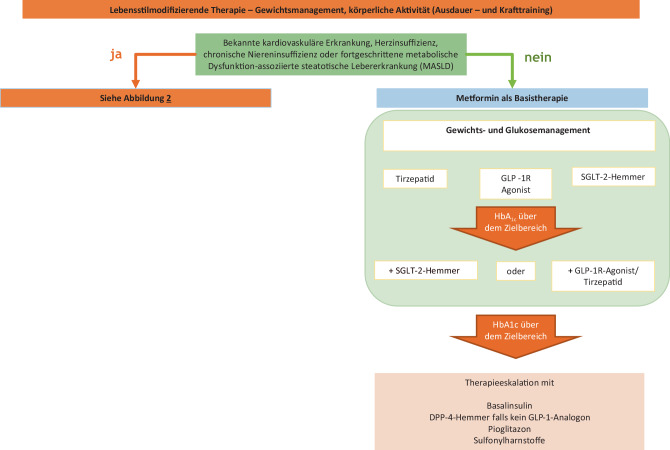
Therapiepfade bei Diabetes mellitus

Möglichkeiten hierfür sind in der Abb. 1 in Anlehnung an die aktuell gültigen Leitlinien der Europäischen bzw. Amerikanischen Diabetesgesellschaft dargestellt.

Große, randomisiert kontrollierte Studien konnten eine signifikante Reduktion kardiovaskulärer Ereignisse bei SGLT-2-Hemmern (Empagliflozin, Canagliflozin, Dapagliflozin) wie auch bei GLP-1-RA (Liraglutid, Dulaglutid und Semaglutid) sowie dem GLP-1R/GIPR-Agonist (Tirzepatid) dokumentieren.

Basierend auf den organprotektiven Daten von Dapagliflozin, Empagliflozin sowie von Semaglutid und Tirzepatid bezüglich Herzinsuffizienz (erhaltene (HFpEF) oder reduzierter Linksventrikelfunktion (HFrEF)) und chronischer Niereninsuffizienz müssen diese Diagnosen bei der Therapieentscheidung unabhängig vom HbA_1c_ zusätzlich berücksichtigt werden.

Wird unter Metformin der individuell festgelegte HbA_1c_-Zielwert nicht erreicht, so wird bei der erforderlichen Therapieeskalation die Berücksichtigung kardiovaskulärer/renaler Komorbiditäten und des Körpergewichts empfohlen.

Das HbA_1c_ stellt die primäre Richtgröße der glykämischen Einstellung dar. Nüchternglukose und postprandiale Glukose sowie Zeit im Zielbereich („time in range“ [TIR]) stellen weitere Richtgrößen dar, die zur Beurteilung der Stoffwechseleinstellung herangezogen werden können. Natürlich müssen auch der Blutdruck, der Lipidstoffwechsel und weitere Komorbiditäten berücksichtigt und therapiert werden.

### FACT-Box

- Basis jeder Diabetestherapie ist eine lebenslange Lebensstilmodifikation (gesunde Ernährung/Gewichtsreduktion/körperliche Aktivität/Rauchstopp), s. entsprechende Leitlinien.
- Grundsätzlich wird ein HbA_1c_-Zielwert ≤ 6,5 % (48 mmol/mol) empfohlen, sofern das ohne relevante Nebenwirkungen der Therapie und ohne signifikante Hypoglykämien erreicht werden kann.
- Zumindest ein HbA_1c_-Ziel < 7,0 % (53 mmol/mol) sollte zum Organschutz angestrebt werden.
- Metformin, SGLT-2-Hemmer, GLP-1/GIP-RA und GLP-1R/GIPRA nehmen die zentrale Rolle in der medikamentösen Behandlung ein.
- Derzeit gibt es für Empagliflozin, Canagliflozin, Dapagliflozin, Liraglutid, Dulaglutid, Semaglutid (oral und subkutan), Tirzepatid positive, substanzspezifische kardiovaskuläre Daten aus placebokontrollierten, randomisierten prospektiven Studien (RCT).
- Bei Herzinsuffizienz (mit erhaltener (HFpEF) oder reduzierter Linksventrikelfunktion (HFmrEF, HFrEF)) sollte unabhängig vom HbA_1c_ ein SGLT-2-Hemmer (Dapagliflozin oder Empagliflozin) eingesetzt werden. Auch für Semaglutid oder Tirzepatid (positive Daten für HFpEF bei Adipositas) liegen Daten bei Herzinsuffizienz vor.
- Bei chronischer Niereninsuffizienz sollten ebenfalls unabhängig vom HbA_1c_ ein SGLT-2-Hemmer und/oder Semaglutid zusätzlich zu anderen, renoprotektiven Medikamenten wie RAAS-Blockern und Finerenon etabliert werden.

Grundsätzlich wird ein HbA_1c_-Zielwert ≤ 6,5 % (48 mmol/mol) empfohlen, sofern ohne relevante Nebenwirkungen (Hypoglykämien) erreichbar.

Zumindest sollte ein HbA_1c_ < 7,0 % (53 mmol/mol) für einen ausreichenden mikrovaskulären, makrovaskulären und zellulären Schutz angestrebt werden.

Neben dem HbA_1c_ stellen die Nüchtern- und die postprandiale Glukose sekundäre Richtgrößen dar. Dementsprechend sollte die Nüchternglukose unter 130 mg/dl (ideal < 110 mg/dl) liegen bzw. die postprandiale Glukose (2 h nach einer Mahlzeit) maximal 180 mg/dl betragen, bei der kontinuierlichen Glukosemessung sollte die Zeit im Zielbereich (TIR) > 70 % der Zeit zwischen 70 mg/dl und 180 mg/dl liegen.

Weiterhin sollte neben der etablierten kardiovaskulären Erkrankung auch das hohe Risiko für eine atherosklerotische, kardiovaskuläre Erkrankung als Indikation für eine Therapie mit GLP-1-RA und oder SGLT-2-Hemmer mit nachgewiesenem kardiovaskulärem Benefit unabhängig vom HbA_1c_ sein.

Alter ≥ 55 Jahre und eines der folgenden Kriterien:

- Linksventrikuläre Hypertrophie
- > 50 % Stenose der Koronarien, Karotiden oder Beinarterien
- eGFR < 60 ml/min/1,73 m^2^
- Albuminurie (UACR > 30 mg/g)

Alternativ kann bei Betroffenen mit Typ-2-Diabetes das kardiovaskuläre Risiko auch anhand des SCORE2-Diabetes errechnet werden.

## Orale antihyperglykämische Therapie

### Metformin

In der Monotherapie wird durch Metformin eine HbA_1c_-Reduktion von ca. 1,5 % bei einer Senkung des Nüchternblutzuckers um 30–40 mg/dl erreicht. Die Metformin-Therapie wird zur Reduktion der Wahrscheinlichkeit gastrointestinaler Unverträglichkeiten vorsichtig mit 2‑mal 500–850 mg pro Tag begonnen und sollte langsam (1- bis 2‑mal wöchentlich) um jeweils 500 mg/Woche bis zu 2000 mg täglich gesteigert werden. Generell ist auch bei übergewichtigen, geriatrischen Menschen mit Diabetes mellitus Typ 2 eine initiale Therapie mit Metformin zu empfehlen. Der appetithemmende und damit gewichtsreduzierende Effekt von Metformin kann aber gerade im geriatrischen Kollektiv aufgrund der Gefahr einer Malnutrition unerwünscht sein (s. Geriatrieleitlinie). Als Kontraindikationen für die Metformin-Therapie gelten eine schwere Einschränkung der Nierenfunktion (GFR < 30 ml/min/1,73 m^2^), dekompensierte Lebererkrankungen, akute Pankreatitis, Alkoholismus, Malnutrition, eine dekompensierte Herzinsuffizienz und/oder andere hypoxische Situationen. Metformin sollte bei Menschen mit eGFR-Werten zwischen 30 und 45 ml/min/1,73 m^2^ bei Fehlen von anderen Risikofaktoren für Laktatazidosen in einer Dosierung von 1000 mg, täglich aufgeteilt auf 2 Dosen, reduziert werden. Die glomeruläre Filtrationsrate sollte mithilfe einer entsprechenden Formel (CKD-EPI) evaluiert und zumindest alle 3 bis 6 Monate kontrolliert werden. Falls die eGFR unter 30 ml/min/1,73 m^2^ abfällt, muss Metformin abgesetzt werden. Bei Operationen, akuten schweren Erkrankungen (z. B.: schwere Infektionen) sowie auch bei Diarrhö und Exsikkose und der Applikation von Kontrastmittel sollte Metformin ebenso vorübergehend pausiert werden (s. Krankenhausleitlinie). Da es unter einer Therapie mit Metformin durch Hemmung des Cubilinrezeptors zu einem Vitamin‑B_12_-Mangel kommen kann, wird empfohlen, die Vitamin‑B_12_-Spiegel jährlich im Rahmen der regelmäßigen Blutabnahmen zu kontrollieren und bei Mangel eine Substitution mit einem Hochdosispräparat (das durch passive Diffusion ausreichend wirkt) durchzuführen.

### SGLT-2-Inhibitoren

Der Natrium-Glukose-Kotransporter (SGLT-2) ist verantwortlich für den größten Teil der Glukoserückresorption im proximalen Tubulus der Niere. Die SGLT-2-Inhibitoren bewirken daher eine Glukosurie und in Folge eine Reduktion der Hyperglykämie. Die Wirkung der SGLT-2-Hemmer ist unabhängig von Insulin. Die in Österreich aktuell verfügbaren Substanzen sind Dapagliflozin, Empagliflozin. Prinzipiell können SGLT-2-Hemmer in jeder Kombination eingesetzt werden. Neben der Blutzuckersenkung (das HbA_1c_ sinkt um 0,5–1 %) kommt es zu einer Senkung des Blutdruckes (2–4/1–2 mm Hg) und zu einer Gewichtsabnahme (−2–3 kg). Neben der glukosurischen Wirkung dieser Medikamentenklasse ergibt sich auch ein diuretischer und gering kaliumsenkender Effekt.

Empagliflozin und Dapagliflozin bewirken eine signifikante Reduktion sowohl kardiovaskulärer als auch renaler Endpunkte. Sowohl für Dapagliflozin als auch für Empagliflozin konnte bei Betroffenen mit Herzinsuffizienz (erhaltene [HFpEF] – oder reduzierte Linksventrikelfunktion [HFmrEF, HFrEF]) eine signifikante Reduktion der Hospitalisationen aufgrund von Herzinsuffizienz gezeigt werden.

Daten aus der DAPA-CKD-Studie (Dapagliflozin), der EMPA-Kidney- (Empagliflozin) als auch aus der CREDENCE-Studie (Canagliflozin) unterstützen die Empfehlung, dass bei chronischer Niereninsuffizienz, SGLT-2-Hemmer mit Evidenz für Reduktion der Progression der chronischen Niereninsuffizienz eingesetzt werden sollen. Empagliflozin kann bis zu einer eGFR > 20 ml/min/1,73 m^2^ und Dapagliflozin bis zu einer eGFR > 25 ml/min/1,73 m^2^ laut Zulassung neu begonnen werden. Aktuelle internationale Leitlinien (KDIGO) empfehlen selbst bei einer eGFR < 20 ml/min/1,73 m^2^ das Fortführen der bereits etablierten SGLT-2-Hemmer-Therapie.

Unter Therapie mit SGLT-2-Inhibitoren wurde das Auftreten von potenziell vital bedrohlichen euglykämischen Ketoazidosen berichtet. Folgende, mögliche Risikofaktoren sind bisweilen bekannt: Infektionen, Low-Carbohydrate oder ketogene Diät, endogener Insulinmangel, unterdosierte Insulintherapie, Reduktion/Absetzen einer laufenden Insulintherapie, Operationen, Absetzen von oralen Insulinsekretagoga, Diabetes mellitus Typ 1 und Alkoholmissbrauch. Entsprechend einer Warnung der EMA sollten zur Risikominimierung des Auftretens einer möglichen Ketoazidose unter den SGLT-2-Inhibitoren generell einige Vorsichtsmaßnahmen eingehalten werden. Bei Verordnung des Medikamentes muss eine Aufklärung über die Symptome der Ketoazidose (z. B. Polyurie, Polydipsie, Lethargie, unklare Gewichtsabnahme, Bauchschmerzen) erfolgen. Sollte sich eine Ketoazidose bestätigen, müssen die SGLT-2-Inhibitoren sofort abgesetzt werden. Bei Menschen mit Risikofaktoren für eine Ketoazidose (geringe Insulinsekretionsreserve; Erkrankungen, welche die Nahrungsmitteleinnahme reduzieren; schwere Dehydratation, plötzliche Insulindosisreduktionen, Operationen oder Alkoholabusus) sollten die SGLT-2-Inhibitoren vorsichtig eingesetzt bzw. vorübergehend pausiert werden. Generell sollen SGLT-2-Inhibitoren bei Operationen und/oder schweren Erkrankungen passager pausiert werden (s. Krankenhausleitlinie).

### GLP-1-Rezeptoragonisten

„Glucagon-like peptide-1“(GLP-1)-Rezeptoranaloga (Liraglutid, Semaglutid, Dulaglutid) führen zu einer postprandialen Steigerung der pankreatischen Insulinsekretion, Hemmung der Glukagonfreisetzung und der Magenentleerung sowie Auslösung eines Sättigungsgefühls durch Stimulation des Sättigungszentrums im Gehirn. Die Applikation erfolgt subkutan 1‑mal täglich bzw. 1‑mal wöchentlich. Neben effektiver Blutzuckerreduktion sind die fehlende Hypoglykämieneigung und die Gewichtsreduktion festzuhalten. Gastrointestinale Beschwerden (Übelkeit, Erbrechen) treten häufiger als unter Placebo auf. Im Rahmen der LEADER-Studie bewirkte Liraglutid eine signifikante Senkung des präspezifizierten kardiovaskulären Endpunktes, wobei dies maßgeblich auf einer signifikanten Reduktion kardiovaskulärer Todesfälle basiert. Semaglutid konnte in der SUSTAIN-6-Studie eine signifikante Reduktion des kombinierten primären Endpunktes (kardiovaskulärer Tod, nichttödlicher Myokardinfarkt, nichttödlicher Insult) erreichen. Die SOUL-Studie konnte nun auch für das oral verfügbare Semaglutid eine Reduktion kardiovaskulärer Endpunkte demonstrieren. Im Rahmen der FLOW-Studie reduzierte Semaglutid bei bereits vorliegender Nephropathie das Auftreten des kombinierten, renalen Endpunktes verglichen mit Placebo um 24 %. Eine Verbesserung der Gehstrecke bei pAVK konnte durch Semaglutid in der STRIDE-Studie erreicht werden. Auch für die Behandlung der Herzinsuffizienz liegen positive, klinische Endpunktdaten vor. In der REWIND-Studie (Dulaglutid) konnte der primäre Endpunkt signifikant um relative 12 % reduziert werden.

### Duale Agonisten GLP-1/GIP

Bei Menschen mit Typ-2-Diabetes und Metformin-Vortherapie fand sich unter Tirzepatid im Vergleich zu Insulin Degludec eine stärkere Reduktion des HbA_1c_-Wertes, eine Reduktion des Körpergewichts und ein niedrigeres Hypoglykämierisiko [5]. Trotz einer längeren Diabetesdauer bewirkte Tirzepatid in der SURPASS 3-Studie bei bis zu 48 % der Teilnehmenden ein HbA_1c_ < 5,7 %. Auch im direkten Vergleich mit Semaglutid zeigte Tirzepatid hinsichtlich der HbA_1c_-Senkung und der Gewichtsreduktion einen größeren Effekt. Tirzepatid zeigte sich in der SURPASS CVOT gegenüber Dulaglutid bezüglich des kombinierten kardiovaskulären Endpunkts nicht unterlegen [6].

### DPP-IV-Hemmer

Dipeptidylpeptidase-IV-Inhibitoren (Sitagliptin, Vildagliptin, Saxagliptin, Linagliptin) als Abbauhemmer des körpereigenen GLP‑1 führen zu einer glukoseabhängigen Steigerung der pankreatischen Insulinsekretion und Hemmung der Glukagonfreisetzung. Diese Substanzen zeigen keine Hypoglykämieneigung und sind gewichtsneutral. Sie werden in der Monotherapie als auch in Kombination mit Metformin (primär), anderen oralen antihyperglykämischen Medikamenten oder aber in Tripelkombination eingesetzt. In Kombination mit Metformin wird eine substanzeigene HbA_1c_-Senkung von ca. 0,8 % beobachtet.

Endpunktstudien belegen die kardiovaskuläre Sicherheit der DPP-IV-Hemmer, ein substanzspezifischer kardiovaskulärer Nutzen konnte aber nicht nachgewiesen werden.

### Pioglitazon

Pioglitazon erhöht die Insulinsensitivität als Ligand der nukleären Hormonrezeptorfamilie PPAR‑ϒ über die Regulation der Expression verschiedener insulinempfindlicher Gene. Im Fettgewebe erfolgt eine verstärkte Differenzierung von Präadipozyten zu Adipozyten und damit eine Beeinflussung der metabolischen und endokrinen Aktivität. Die Insulinsensitivität in Leber, Skelettmuskel und im Fettgewebe nimmt zu. In Abhängigkeit vom Ausgangs-HbA_1c-_-Wert und der Dosierung reduzieren Glitazone den HbA_1c_-Wert um etwa 1,5 %. Zu den Nebenwirkungen der PPAR-γ-Agonisten zählen Gewichtszunahme und verstärkte Ödemneigung auf Basis von Flüssigkeitsretention. Kontraindikation für die Glitazontherapie ist die Herzinsuffizienz (wegen Flüssigkeitsretention durch erhöhte Natriumrückresorption). Pioglitazon selbst hat keine direkten negativen, kardialen Effekte. Bei postmenopausalen Frauen wurde eine Steigerung traumatischer Knochenbrüche beobachtet. In der PROACTIVE-Studie hat Pioglitazon den sekundären Endpunkt (MACE) signifikant gesenkt. Pioglitazon hat auch signifikante Effekte auf die Reduktion von erneuten kardiovaskulären Ereignissen bei Menschen mit erhöhtem Diabetesrisiko gezeigt (IRIS-Studie).

### Sulfonylharnstoffe

Sulfonylharnstoffe (Gliclazid, Glimepirid) stimulieren die pankreatische Insulinsekretion und resultieren in einer mittleren zu erwartenden HbA_1c_-Reduktion um 1,5 %. Zu den klinisch relevanten Nebenwirkungen zählt das erhöhte Hypoglykämierisiko. Gliclazid hat ein niedrigeres Hypoglykämierisiko im Vergleich zu den anderen Sulfonylharnstoffen. Eine Gliclazid-basierte große randomisierte Outcome-Studie konnte eine signifikante Reduktion mikrovaskulärer Ereignisse zeigen (ADVANCE). In den letzten Jahren wurden Sulfonylharnstoffe in Zusammenhang mit einem erhöhten kardiovaskulären Risiko gebracht, welches in Metaanalysen für die angeführten Sulfonylharnstoffe nicht bestätigt werden konnte. Die Resultate der Metaanalysen konnten in der randomisiert, prospektiven CAROLINA-Studie bestätigt werden. So wurde für Glimepirid kein Signal für ein erhöhtes kardiovaskuläres Risiko beobachtet.

**Tab. 1:** Überblick über die blutzuckersenkende Therapie

Klasse	HbA_1c_ (in %)	Hypoglykämie	Vorteile	Klinisch relevante Nebenwirkungen
Metformin	1–2	Nein	Gewichtsneutralität, Reduktion makrovaskulärer Ereignisse	Gastrointestinale Nebenwirkungen Laktatazidose
SGLT-2-Hemmer	0,5–1	Nein	Empagliflozin, Dapagliflozin reduzieren kardiovaskuläre Ereignisse, positive Daten bei HFpEF und HFrEF, Nephroprotektion Gewichtsreduktion	Genitale Infekte, selten Auslöser euglykämischer Ketoazidosen
GLP-1-Rezeptoragonisten	1–2	Nein	Gewichtsreduktion Reduktion kardiovaskulärer Ereignisse unter Liraglutid, Dulaglutid und Semaglutid, Nephroprotektion	Gastrointestinale Nebenwirkungen, subkutane Injektion
GLP-1R – GIPR-Agonisten	2–2,3	Nein	Gewichtsreduktion Reduktion, kardiovaskulärer Ereignisse, Nephroprotektion	Gastrointestinale Nebenwirkungen, subkutane Injektion
Pioglitazon	1–2	Nein	Reduktion kardiovaskulärer Ereignisse im sekundären Endpunkt	Gewichtszunahme, periphere Ödeme, Frakturen bei Frauen
DPP-4-Hemmer	0,5–1	Nein	Gewichtsneutral Kardiovaskulär sicher	Moderate Wirksamkeit
Sulfonylharnstoffe	1–2	Ja	Rasche Blutzuckersenkung Kardiovaskulär sicher	Mögliche Gewichtszunahme, Hypoglykämien
Insulin	> 2	Ja	Keine Dosisobergrenze Flexible Therapieführung	Gewichtszunahme, Hypoglykämie

### Insuline

Nach Ausschöpfung der nicht insulinbasierten, blutzuckersenkenden Therapieprinzipien stellt die Basalinsulintherapie eine einfache und gleichzeitig auch sichere Möglichkeit für den Einstieg in eine Insulintherapie dar. Kann unter dieser Therapie das individuell festgelegte Therapieziel nicht erreicht werden, so sollte je nach Wünschen und Bedürfnissen des Menschen mit Diabetes mellitus Typ 2 eine Intensivierung der Therapie mithilfe eines zusätzlich verabreichten, prandialen Insulins erfolgen (s. Leitlinie Diagnose und Therapie des Diabetes mellitus Typ 1).

## Evidenzlage

Der epidemiologische Zusammenhang zwischen dem Ausmaß der Hyperglykämie und dem Auftreten von Organkomplikationen ist absolut gesichert.

Die zentrale Evidenz der UKPDS ist, dass eine intensivierte Therapie mit Insulin oder Sulfonylharnstoffen einer konventionellen diätetischen Therapie im Hinblick auf Komplikationen überlegen ist, wobei eine Verbesserung des HbA_1c_ um 0,9 % erreicht wurde [7].

Ein spezifischer Substanzvorteil zeigte sich für Metformin bei Menschen mit Adipositas [8]. In dieser Gruppe wurden Myokardinfarkte sowie Diabetes-assoziierte Mortalität und Gesamtmortalität signifikant gesenkt. Die Follow-up-Untersuchung der UKPDS-Population legt nahe, dass durch intensivierte Therapie langfristig die Gesamtmortalität gesenkt werden kann [8]. Bei Menschen mit neuer Diabetesmanifestation konnte in UKPDS die Existenz eines „metabolischen Gedächtnisses“ nachgewiesen werden, ein um 2 % höherer HbA_1c_-Wert war mit einer um 86 % gesteigerten Mortalität assoziiert.

UKPDS [8], ADVANCE [9], ACCORD [10, 11] zeigen, dass eine gute Blutzuckerkontrolle durch intensivierte Therapiestrategien möglichst unmittelbar nach Diagnosestellung erreicht und ohne schwere Hypoglykämien und exzessive Gewichtszunahme aufrechterhalten werden sollte. Langzeitbeobachtungen der Studien haben gezeigt, dass eine kardiovaskuläre Risikoreduktion von ca. 20 % möglich ist [12]. In diesen Studien hatten die Patient:innengruppen mit den tiefsten HbA_1c_-Werten auch die niedrigste Mortalitätsrate pro Jahr.

### Kardiovaskuläre Endpunktstudien

Die EMPA-REG-Outcome-Studie [13] zeigte eine signifikante Reduktion des primären Endpunktes (kardiovaskulärer Tod, nichttödlicher Myokardinfarkt oder nichttödlicher Insult) bei kardiovaskulär vorerkrankten Patient:innen mit Diabetes durch Empagliflozin verglichen mit Placebo. Kardiovaskulärer Tod und Gesamtmortalität wurden bei Patient:innen mit einer atherosklerotischen Erkrankung signifikant gesenkt. Die Hospitalisierungsrate für Herzinsuffizienz sank unter Empagliflozin um 35 %, das Auftreten von nichttödlichem Herzinfarkt, instabiler Angina pectoris und nichttödlichem Schlaganfall wurde hingegen nicht signifikant beeinflusst.

Dapagliflozin wurde in der DECLARE-TIMI 58-Studie an 17.160 Teilnehmenden untersucht, wobei 10.186 Menschen am Beginn der Studie keine kardiovaskuläre Erkrankung aufwiesen. Es wurde eine signifikante Reduktion des kombinierten Endpunktes kardiovaskulärer Todesfälle und Hospitalisationen aufgrund von Herzinsuffizienz registriert.

Hinsichtlich der GLP-1-Analoga liegen für Liraglutid, Dulaglutid und Semaglutid positive Daten aus einer kardiovaskulären Endpunktstudie vor. In der LEADER-Studie bewirkte Liraglutid eine signifikante Reduktion kardiovaskulärer Ereignisse (inklusive Todesfälle). Dulaglutid zeigte ebenso einen signifikanten kardiovaskulären Benefit [14, 15].

Für Semaglutid bestätigt die SUSTAIN-6-Studie die positiven kardiovaskulären Effekte. Der primäre Endpunkt trat in der Semaglutid-Gruppe signifikant geringer auf, wobei nichttödliche Schlaganfälle in der Semaglutid-Gruppe signifikant reduziert wurden. Semaglutid bewirkte weiters in der STRIDE-Studie eine signifikante Verbesserung der Gehstrecke bei Menschen mit bekannter pAVK [16].

In der SOUL-Studie konnte das orale Semaglutid eine signifikante Reduktion des 3‑Punkt-MACE erreichen [17].

Unter Semaglutid kam es vermehrt zu einer Verschlechterung einer bestehenden diabetischen Retinopathie (Glaskörperblutung, Erblindung, intravitreale Injektionen oder Photokoagulation) [18]. Das Risiko trat nur bei Menschen mit hohen HbA_1c_-Ausgangswerten und bei sehr rascher Blutzuckersenkung und vorbestehender diabetischer Retinopathie auf. Eine weitere, mögliche, sehr seltene Nebenwirkung von Semaglutid (bis zu 1 von 10.000 behandelten Personen) ist die nichtarteriitische anteriore ischämische Optikusneuropathie. Patient:innen, die während der Behandlung plötzlich Sehverlust oder eine rasch zunehmende Verschlechterung der Sehfähigkeit bemerken, sollten umgehend ärztlichen Rat einholen. Wird eine NAION diagnostiziert, ist die Behandlung mit Semaglutid umgehend abzubrechen.

Die Datenlage für Pioglitazon ist hinsichtlich einer möglichen kardiovaskulären Prävention ebenso weitgehend positiv. Für Pioglitazon existiert mit PROACTIVE eine positive Endpunktstudie [19], die hinsichtlich des sekundären Endpunktes schwerer kardiovaskulärer Ereignisse insgesamt und besonders für die Subgruppen der Patient:innen mit vorangegangenem Myokardinfarkt [20] oder Schlaganfall [21] deutliche Vorteile zeigt.

Für Insulin Glargin (ORIGIN-Studie) und Insulin Degludec (DEVOTE-Studie) konnte die kardiovaskuläre Sicherheit dieser Langzeitinsulinanaloga gezeigt werden [22, 23].

### Herzinsuffizienz

Sowohl Dapagliflozin, Empagliflozin als auch Canagliflozin haben in den jeweiligen Endpunktstudien deutliche Reduktionen in der Hospitalisierung aufgrund einer Herzinsuffizienz gezeigt.

In die DAPA-HF-Studie wurden Personen mit vorbestehender Herzinsuffizienz mit reduzierter Auswurffraktion eingeschlossen. In der Dapagliflozin-therapierten Gruppe zeigte sich eine signifikante Reduktion des primären Endpunktes (Verschlechterung der Herzinsuffizienz – dies war entweder eine Hospitalisierung oder dringliche Visite mit intravenöser Herzinsuffizienztherapie – oder kardiovaskulärer Tod) [24].

In der EMPEROR-Reduced-Studie wurde Empagliflozin 10 mg mit Placebo als Add-on zur etablierten, leitliniengerechten Herzinsuffizienztherapie untersucht. Es konnte der primäre Endpunkt (Hospitalisierungsrate aufgrund von Herzinsuffizienz oder kardiovaskulärer Tod) durch die Gabe von Empagliflozin signifikant gesenkt werden [25]. Im direkten Vergleich lag die Ereignisrate für den primären Endpunkt in der EMPEROR-Studie höher als in DAPA-HF, was sich letztlich auch durch die Tatsache erklären lässt, dass die Teilnehmenden in der EMPEROR-Studie durchwegs fortgeschrittenere Stadien der Herzinsuffizienz aufwiesen.

Die Emperor-Preserved-Studie untersuchte Menschen, deren linksventrikuläre Auswurffraktion (LVEF) > 40 % war. Letztlich bewirkte Empagliflozin eine signifikante Reduktion des primären Endpunktes (kardiovaskulärer Tod oder Hospitalisation aufgrund von Herzinsuffizienz) um 21 % [24].

Analog zur Emperor-Preserved-Studie zeigte sich für Dapagliflozin in der DELIVER-Studie ebenfalls ein positiver Effekt bei Patient:innen mit einer LVEF > 40 %. Über einen Nachbeobachtungszeitraum von 2,3 Jahren konnte der primäre Endpunkt (Verschlechterung der Herzinsuffizienz oder kardiovaskulärer Tod) signifikant reduziert werden [26].

**Abb. 2:**
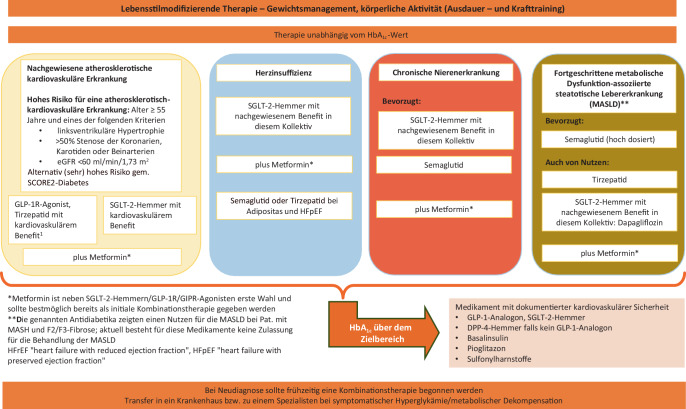
Therapiepfade bei Diabetes mellitus

Diese Daten ergänzen und verstärken die Empfehlung, dass bei vorbestehender Herzinsuffizienz unabhängig von der LVEF und unabhängig vom HbA_1c_ ein SGLT-2-Hemmer mit Evidenz zur Reduktion von Herzinsuffizienz (s. Abb. 2) eingesetzt werden sollte.

**Abb. 3:**
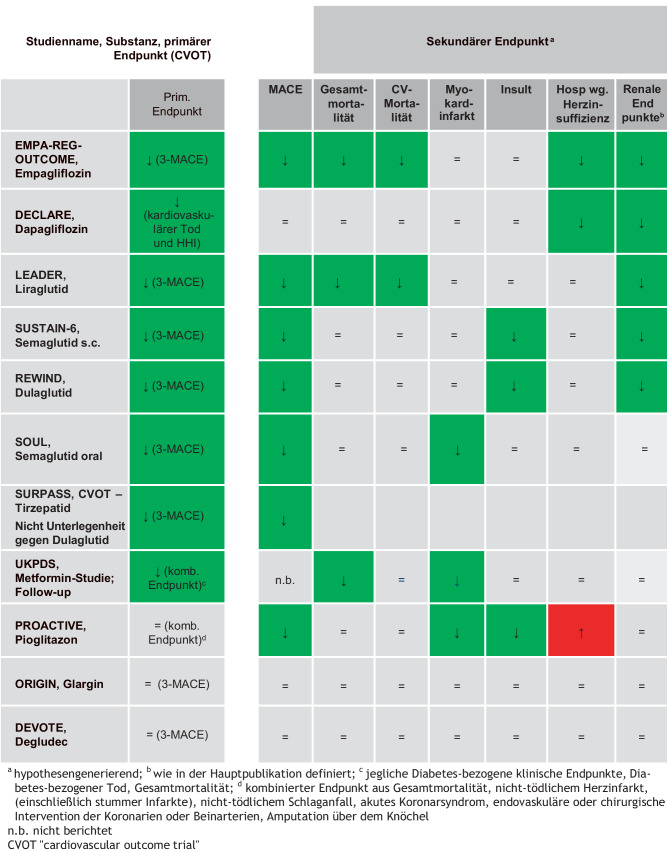
Evidenz zu kardiovaskulärer Sicherheit und Vorteil antidiabetischer Substanzen aus randomisierten, placebokontrollierten Studien bei Patient:innen mit T2DM

**Abb. 4:**
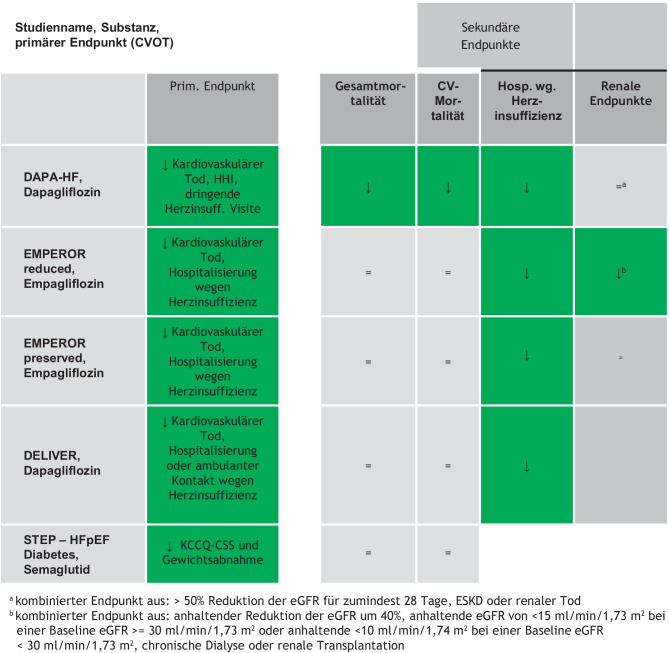
Endpunktstudien bei Patient:innen mit Herzinsuffizienz

**Abb. 5:**
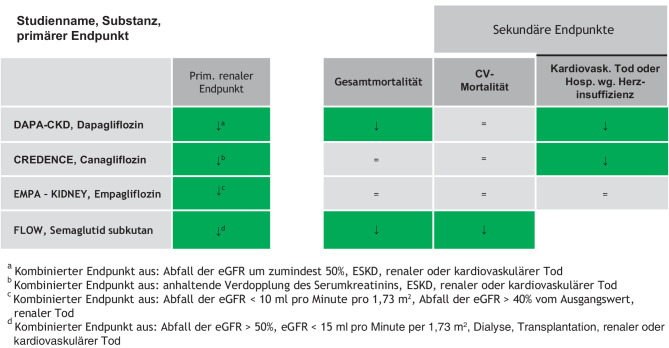
Endpunktstudien bei Patient:innen mit chronischer Nierenerkrankung

Auch für Semaglutid liegen in der Indikation Herzinsuffizienz mit erhaltener oder leicht reduzierter Linksventrikelfunktion positive Daten vor. In der STEP-HFpEF DM-Studie bewirkte Semaglutid eine signifikante Reduktion der klinischen Symptome der Patient:innen gemessen mit dem Kansas City Cardiomyopathy Questionnaire clinical summary. Die Resultate der STEP HFpEF DM-Studie sprechen für einen Effekt, welcher unabhängig von den SGLT-2-Hemmern ist und somit eine therapeutische Ergänzung darstellen dürfte [27]. Tirzepatid reduzierte bei Menschen mit Adipositas (48 % mit Typ-2-Diabetes) und Herzinsuffizienz mit erhaltener Linksventrikelfunktion (HFpEF) den koprimären Endpunkt kardiovaskulärer Tod oder ein Ereignis durch eine symptomatisch verschlechterte Herzinsuffizienz signifikant [28].

### Diabetische Nierenerkrankung

In der DAPA-CKD-Studie wurden 4304 Menschen mit chronischer Niereninsuffizienz (eGFR 25–75 ml/min/1,73 m^2^ und einer Albumin-Kreatinin-Ratio von 200–5000 mg/g) untersucht. Die Prävalenz des Diabetes lag bei 67,5 %. Die Studie wurde nach einer medianen Beobachtungszeit von 2,4 Jahren aufgrund der positiven Effekte von Dapagliflozin gestoppt. Die Gabe von Dapagliflozin konnte den primären Endpunkt (Abnahme der eGFR um mindestens 50 %, terminale Niereninsuffizienz oder Tod aufgrund einer kardiovaskulären oder renalen Ursache) signifikant reduzieren. Das Vorliegen eines Diabetes mellitus hatte keinen signifikanten Einfluss auf die positiven Effekte von Dapagliflozin hinsichtlich der gewählten Endpunkte [29].

Die EMPA Kidney-Studie untersuchte 6609 Menschen mit chronischer Niereninsuffizienz (eGFR 20–45 ml/min/1,73 m^2^ oder 45–90 ml/min/1,73 m^2^ und einer Albumin-Kreatinin-Ratio von mindestes 200 mg/g). Während der medianen Nachbeobachtungszeit von 2 Jahren wurde durch die Gabe von Empagliflozin der Endpunkt (Progression der Niereninsuffizienz oder kardiovaskulärer Tod) signifikant gesenkt [30].

Diese Daten unterstützen die Empfehlung, dass bei chronischer Niereninsuffizienz SGLT-2-Hemmer mit Evidenz für die Reduktion der Progression der chronischen Niereninsuffizienz unabhängig von der aktuellen Blutzuckersituation eingesetzt werden sollen (Abb. 2).

Im Rahmen der FLOW-Studie konnte Semaglutid bei bereits vorliegender Nephropathie das Auftreten des kombinierten, renalen Endpunktes, verglichen mit Placebo um 24 % reduzieren. Entgegen den Resultaten der SGLT-2-Hemmer zeigte sich in der Flow-Studie kein anfänglicher Abfall der eGFR in der Semaglutid-Gruppe [31].

### Metabolisch assoziierte steatotische Lebererkrankung (MASLD)

Etwa 75 % der Patient:innen mit Diabetes mellitus Typ 2 haben eine begleitende Fettlebererkrankung unterschiedlichen Grades mit einer hohen Progressionsrate von ca. 5–7 %. Das Vorliegen eines Typ-2-Diabetes ist zudem ein Risikofaktor für die Entwicklung einer fortgeschrittenen Leberfibrose [35]. Fortgeschrittene Stadien einer MASLD im Sinne einer metabolisch assoziierten Steatohepatitis (MASH) werden selten anhand einer Biopsie diagnostiziert, da diese in den wenigsten Fällen indiziert ist. Hingegen kann das Risiko einer signifikanten Fibrose durch nichtinvasive Tests wie einen FIB-4-Score (< 1,3, aber 65 Jahre < 2,0) oder eine Elastographie (< 8 kPa durch vibrationskontrollierte transiente Elastographie [VCTE] oder Scherwellenelastographie [SWE]) weitgehend ausgeschlossen werden. Andererseits sollten bei Patient:innen mit Diabetes mellitus Typ 2 und Vorliegen einer MASLD mit Hinweis auf eine signifikante Fibrose (z. B. ≥ 8 kPa in der Elastographie) für die Blutzuckerkontrolle Medikamente mit nachgewiesenem Nutzen für die Verbesserung der MASH bzw. der Fibrose bevorzugt in Betracht gezogen werden.

Entsprechende Nachweise wurden für Semaglutid in der wöchentlichen Dosis von 2,4 mg in der multizentrischen, randomisierten, doppelblinden, kontrollierten ESSENCE-Studie für eine Population mit MASH und F2 & F3-Fibrose erbracht [32]. In dieser Studie hatten 56 % der eingeschlossenen Patient:innen einen Diabetes mellitus Typ 2. In der publizierten Interimsanalyse mit 800 eingeschlossenen Patient:innen lag der Anteil an Patient:innen mit Verschwinden der MASH ohne Verschlechterung der Fibrose in der Semaglutid-Gruppe nach 78 Wochen um 28,7 %-Punkte höher als unter Placebo (62,9 % vs. 34,2 %, *p* < 0,001). Ebenso war der Anteil an Patient:innen mit einer Reduktion der Fibrose ohne Verschlechterung der MASH um 14,4 %-Punkte höher (36,8 % vs. 22,4 %, *p* < 0,001).

Tirzepatid wurde in der multizentrischen, doppelblinden placebokontrollierten Dosisfindungsstudie (SYNERGY-NASH) in den Dosierungen 5 mg/10 mg/15 mg pro Woche in einer vergleichbaren Population mit MASH und Fibrose F2 und F3, darunter 58 % Patient:innen mit Diabetes mellitus Typ 2, untersucht (Primärer Endpunkt war die Auflösung der MASH ohne Verschlechterung der Fibrose). Nach 52 Wochen trat der primäre Endpunkt bei 157 ausgewerteten Patient:innen für die 3 Dosierungen bei 44 % (5 mg), 56 % (10 mg) bzw. 63 % (15 mg) der Patient:innen ein und damit signifikant häufiger als unter Placebo (10 %; alle *p* < 0,001). Deutlicher Nutzen konnte auch bei der Verbesserung der Fibrose ohne Verschlechterung der MASH nachgewiesen werden [33].

Auch für den SGLT2-Hemmer Dapagliflozin konnte die multizentrische, doppelblinde placebokontrollierte DEAN-Studie aus China einen Nutzen für die MASLD zeigen. Bei 157 Patient:innen mit MASH, darunter 70 (45 %) mit T2DM, 99 (63 %) mit F2 & F3-Fibrose, erhöhte Dapagliflozin den Anteil an Patient:innen mit Verbesserung der MASH ohne Verschlechterung der Fibrose um 23 % (30 % vs. 53 %; *p* = 0,006). Auch die Auflösung der MASH ohne Verschlechterung der Fibrose und die Verbesserung der Fibrose ohne Verschlechterung der MASH wurde signifikant um 15 % bzw. 25 % erhöht [34].

Bei metabolisch assoziierter Leberzirrhose sollte zumindest nach Dekompensation Metformin vermieden werden, ebenso Sulfonylharnstoffe [35]. Inkretinanaloga sind zumindest beim Stadium Child-Pugh A, SGLT-2-Hemmer bei Child-Pugh A und B anwendbar. Das Risiko einer Sarkopenie ist bei diesen Patient:innen zu beachten.

## Interessenkonflikt

M. Clodi gibt folgende Interessenkonflikte an: Vorträge, Studien und Beratung für Novo Nordisk, Elli Lilly, Boehringer Ingelheim, AstraZeneca, Novartis, Daiichi Sankyo, Amgen, Wörwag, Sanofi, Amarin, Roche, Bayer. H. Abrahamian gibt folgende Interessenkonflikte für die letzten 3 Jahre an: Kongressunterstützung durch AstraZeneca und Eli Lilly. F. Aberer gibt folgende Offenlegungen an: erhielt Vortragshonorare von Sanofi-Aventis, Novo Nordisk, AMGEN und Eli Lilly und war Mitglied in Advisory Boards von Bayer, Eli Lilly und Novo Nordisk. A. Bräuer erhielt Einladungen zu Kongressen, Honorare für Beratungs- oder Vortragstätigkeiten bzw. Unterstützungen für Fortbildungsveranstaltungen: AstraZeneca, Boehringer Ingelheim, Eli Lilly, Novo Nordisk, Sanofi-Aventis. K. Aydinkoc-Tuzcu hat von folgenden Unternehmen, die auch fördernde Mitglieder der ÖDG sind, Forschungsunterstützungen und/oder Honorare erhalten: Abbott, AstraZeneca, Bayer Health Care, Bristol Myers Squibb, Germania Pharmazeutika, GlaxoSmithKline Pharma, Eli Lilly, Merck Serono, Merck Sharp & Dohme, Novartis, Novo Nordisk, Pfizer, Roche, Sanofi-Aventis, Takeda. Zusätzlich gibt K. Aydinkoc-Tuzcu an, dass für sie persönlich kein Interessenkonflikt vorliegt. H. Brath hat von folgenden Unternehmen, die z. T. auch fördernde Mitglieder der ÖDG sind, Vortrags- oder Beratungshonorare oder Kongressunterstützungen erhalten: Amarin, Amgen, AstraZeneca, Bayer, Boehringer Ingelheim, Daiichi Sankyo, Eli Lilly, Gilead, MSD, Novartis, Novo Nordisk, Roche, Sandoz, Sanofi, Servier, Wörwag. J. Brix hat von folgenden Unternehmen, die z. T. auch fördernde Mitglieder der ÖDG sind, Forschungsunterstützungen und/oder Honorare erhalten: Abbott, AstraZeneca, Bayer, Boehringer Ingelheim, Dexcom, Eli Lilly, Medtronic, Novo Nordisk. P. Fasching gibt folgende Interessenkonflikte (für die letzten 3 Jahre) an: Honorare für Vorträge, Teilnahme an Advisory Boards und wissenschaftliches Consulting von AstraZeneca, Eli Lilly, Novo Nordisk, Sanofi und Boehringer Ingelheim, sowie Kongressunterstützung von Boehringer Ingelheim und Novo Nordisk und Teilnahme als Primary Investigator (PI) an klinischen Studien von Eli Lilly und Novo Nordisk. A. Feder erhielt Vortragshonorare und/oder Honorare für Beratungstätigkeit von Eli Lilly, AstraZeneca und Bayer. L. Frühwald hat Vortragshonorare und Übernahme von Kongressgebühren erhalten von E. Lilly, Sanofi, Boehringer Ingelheim, Novo Nordisk, Abbott. E. Fröhlich-Reiterer gibt folgende Offenlegungen an: Forschungsunterstützung und Honorare für Vorträge und Advisory Boards von Sanofi, E. Lilly, Medtronic, Merck, Sandoz. J. Harreiter hat von folgenden Unternehmen Forschungsunterstützungen und/oder Honorare erhalten: Amgen, AstraZeneca, Bayer, Boehringer Ingelheim, Novartis, Novo Nordisk, Ultragenyx. S. Hofer hat in den letzten 36 Monaten von folgenden Unternehmen, die auch fördernde Mitglieder der ÖDG sind, Forschungsunterstützungen und/oder Honorare erhalten: E. Lilly, Sanofi und Ypsomed. F. Höllerl hat von AstraZeneca Honorare für Vortragstätigkeiten erhalten. J. Huber hat von folgenden Unternehmen Fortbildungsunterstützung und/oder Honorare für Vorträge und Beratung erhalten: Eli Lilly, Novo Nordisk, Boehringer Ingelheim, AstraZeneca, Sanofi-Aventis, Merck Sharp & Dohme, Daiichi Sankyo, Viatris. Zusätzlich gibt J. Huber an, dass für ihn persönlich kein Interessenkonflikt vorliegt. S. Kaser hat Vortragshonorare bzw. Beraterhonorare erhalten von Boehringer Ingelheim, Novo Nordisk, Eli Lilly, Sanofi, Boehringer Ingelheim, Amarin. A. Kautzky-Willer hat von folgenden Unternehmen, die teils auch fördernde Mitglieder der ÖDG sind, Forschungsunterstützungen und/oder Honorare erhalten: Böhringer Ingelheim, Eli Lilly, Merck Sharp & Dohme, Novartis, Novo Nordisk, Roche, Sanofi-Aventis, Amgen. A. Kietaibl gibt folgende Offenlegungen an: Honorare von Sanofi-Aventis GmbH und Roche Diabetes Care Austria GmbH erhalten. Kongressunterstützung durch Novo Nordisk, Eli Lilly GmbH, Boehringer Ingelheim und ÖDG. G. Köhler gibt folgende Interessenkonflikte an: Honorare von Amgen, Eli Lilly, Bayer, Böhringer Ingelheim, Daiichi Sankyo, Dexcom, Novartis, Novo Nordisk, Sanofi-Aventis GmbH und Roche Diabetes Care erhalten. S.E. Hofer gibt folgende Interessenkonflikte (für die letzten 3 Jahre) an: Honorare für Vorträge und Teilnahme an Advisory Boards erhalten von Sanofi, E. Lilly, Ypsomed. M. Leutner hat von folgenden Unternehmen, die auch tlw. fördernde Mitglieder der ÖDG sind, Forschungsunterstützungen und/oder Honorare erhalten: Abbott, AstraZeneca, Bayer, Boehringer Ingelheim, Daiichi Sankyo, Sobi, Novartis, Novo Nordisk. B. Ludvik gibt folgende Interessenkonflikte an: Honorare für Advisory Boards, Vorträge und Forschungsunterstützung von: Amgen, AstraZeneca, Boehringer Ingelheim, Daiichi Sankyo, Eli Lilly, Novo Nordisk. J.K. Mader gibt folgende Interessenkonflikte an: Advisory Boards von Abbott Diabetes Care, Becton-Dickinson, Biomea Fusion, Dexcom, Eli Lilly, Embecta, Insulet, Medtronic, myLife, Novo Nordisk A/S, Pharmasens, Roche Diabetes Care, Sanofi-Aventis, Tandem und Viatris; Honorare für Vortragstätigkeit: A. Menarini Diagnostics, Abbott Diabetes Care, Dexcom, Eli Lilly, Medtrust, MSD, Novo Nordisk A/S, Roche Diabetes Care, Sanofi, Viatris und Ypsomed. Gesellschafterin: decide Clinical Software GmbH und elyte Diagnostics. V. Parzer hat Honorare für Vortragstätigkeit/Kongressunterstützung von Eli Lilly und Novo Nordisk erhalten. B. Paulweber hat von folgenden Unternehmen, die z. T. auch fördernde Mitglieder der ÖDG sind, Forschungsunterstützungen und/oder Honorare erhalten: Amgen, AstraZeneca, Bayer, Boehringer Ingelheim, Eli Lilly, MSD, Novo Nordisk, Pfizer, Sanofi und Sobi. B. Rami-Merhar hat in den letzten 36 Monaten von folgenden Firmen Honorare/Forschungsgelder erhalten: Eli Lilly, Medtronic, Insulet, Abbott, Sanofi. G. Rega-Kaun gibt folgende Interessenkonflikte (für die letzten 3 Jahre) an: Honorare für wissenschaftliche Vorträge und Beratung: Amgen, Bayer Austria, Boehringer, Daiichi Sankyo, Eli Lilly, Novartis, Novo Nordisk, Sanofi, Ultragenyx und Viatris. Sowie Kongressunterstützung von Eli Lilly, Novartis, Novo Nordisk und Ultragenyx. M. Resl hat von folgenden Firmen Honorare und Forschungsgelder erhalten: Eli Lilly, Abbott, Boehringer Ingelheim, Roche, Bayer. M. Riedl hat von Boehringer Ingelheim und Novo Nordisk Honorare und Forschungsgelder erhalten. M. Roden gibt folgende Interessenkonflikte an: Honorare für wissenschaftliche Vorträge und wissenschaftliche Beratung von AstraZeneca, Boehringer Ingelheim, Echosens, Eli Lilly, Madrigal, Merck-MSD, Novo Nordisk, Synlab und Target RWE. M. Roden führt „investigator-initiated studies“ mit Unterstützung von Boehringer Ingelheim, Novo Nordisk und Sanofi und das Deutsche Diabetes Zentrum (DDZ) durch. C.H. Saely hat Forschungsförderungen bzw. Sprecher- und Beraterhonorare von AstraZeneca, Böhringer Ingelheim, Lilly, Merck, MSD, Mylan, Novartis, Sanofi-Aventis, Viatris erhalten. C. Schelkshorn hat Sprecher- und Beraterhonorare sowie Forschungsförderungen von Novo Nordisk, Boehringer Ingelheim, Sanofi, Abbott Diabetes, Lilly, Dexcom, Ypsomed, Roche erhalten. H. Sourij hat von folgenden Unternehmen Honorare für Vorträge oder Beratertätigkeit erhalten: Amarin, Amgen, AstraZeneca, Boehringer Ingelheim, Bayer, Daiichi Sankyo, Eli Lilly, Novo Nordisk, Novartis, Sanofi, Sobi. L. Stechemesser hat für Vortragstätigkeit und Forschungszusammenarbeit mit Abbott, Amarin, Amgen, AstraZeneca, Bayer, Boehringer Ingelheim, CSL Vifor, Daiichi Sankyo, Dexcom, Eli Lilly, Medtronic, Mylan, Novartis, Novo Nordisk, Roche Diabetes Care, Sanofi-Aventis Honorare erhalten. H. Stingl hat von folgenden Unternehmen, die teils auch fördernde Mitglieder der ÖDG sind, Honorare für Vorträge und/oder Beratertätigkeit bzw. Fortbildungsunterstützung erhalten: Abbott, Amarin, Amgen, AstraZeneca, Bayer, Boehringer Ingelheim, Daiichi Sankyo, Eli Lilly, Novo Nordisk, Novartis, Sanofi-Aventis, Viatris. T. Stulnig hat von folgenden Unternehmen Reiseunterstützung, Honorare für Vorträge, Beratertätigkeit oder Forschungsunterstützungen erhalten: 89bio, Akcea, Amarin, Amgen, AstraZeneca, Boehringer Ingelheim, Eli Lilly, Novo Nordisk, Sanofi. M. Tauschmann war Mitglied von Advisory Boards für Sanofi und Abbott, erhielt Vortragshonorare von Eli Lilly, Medtronic, Sanofi und Ypsomed und Unterstützung bei Konferenzteilnahme durch die KelCon GmbH. H. Toplak hat von folgenden Unternehmen Honorare für Vorträge und Forschungsunterstützungen erhalten: Vorträge für Böhringer Ingelheim und Novo Nordisk sowie Forschungsunterstützung; klinische Studien für Boehringer Ingelheim und Novo Nordisk. G. Treiber gibt folgende Interessenkonflikte an: Honorare für Vorträge und Reisekostenunterstützung für Kongressteilnahme erhalten von Medtronic AT, Sanofi, E. Lilly, Novo Nordisk, Bayer Austria, ÖDG, Österreichische Akademie der Ärzte. A. Vonbank gibt folgende Interessenkonflikte an: Fortbildungsunterstützungen bzw. Honorare für Forschungstätigkeit, Beratungstätigkeit oder Vorträge: Eli Lilly, Boehringer Ingelheim, Amarin, Daiichi Sankyo, Novo Nordisk, Sanofi-Aventis, AstraZeneca, Amgen, Bayer, LKH Feldkirch. T.C. Wascher gibt folgende Interessenkonflikte an: Forschungsunterstützungen und/oder Honorare von Amgen, AstraZeneca, Bayer Austria, Eli Lilly, MSD, Novartis, Novo Nordisk, Sanofi, Daiichi Sankyo. R. Weitgasser hat für Vortragstätigkeit und Forschungszusammenarbeit mit Spar, Eli Lilly, Novo Nordisk, Daiichi Sankyo und Boehringer Ingelheim Honorare erhalten. Y. Winhofer gibt folgende Interessenkonflikte (für die letzten 3 Jahre) an: Honorare für Vorträge, Teilnahme an Advisory Boards, wissenschaftliches Consulting und Kongressunterstützung von folgenden Unternehmen: Abbott, Amarin, AstraZeneca, Bayer Austria, Boehringer Ingelheim Daiichi Sankyo, Dexcom, Eli Lilly, MSD, Novartis, Novo Nordisk, Roche Diabetes Care, Sanofi-Aventis. S. Zlamal-Fortunat hat in den letzten 36 Monaten Honorare für Vorträge und Advisory Boards von Novo Nordisk, Sanofi, Johnson & Johnson, Bayer, Amarin, AstraZeneca, Boehringer Ingelheim, Medtronic, Daiichi Sankyo und Novartis erhalten. H. Drexel, M. Fritsch, B. Föger, C. Francesconi, F. Hoppichler, S. Huber, M. Lechleitner, T. Pieber, R. Prager, E. Pucher, C. Ress, G. Schernthaner und Th. Steinmaurer geben an, dass kein Interessenkonflikt besteht.

## References

[1] Honigberg MC, et al. Cardiovascular and Kidney Outcomes Across the Glycemic Spectrum: Insights From the UK Biobank. J Am Coll Cardiol. 2021;78(5):453–64. 34015477 10.1016/j.jacc.2021.05.004 PMC8324525

[2] Lincoff AM, et al. Semaglutide and Cardiovascular Outcomes in Obesity without Diabetes. N Engl J Med. 2023;389(24):2221–32. 37952131 10.1056/NEJMoa2307563

[3] Jastreboff AM, et al. Tirzepatide for Obesity Treatment and Diabetes Prevention. N Engl J Med. 2025;392(10):958–71. 39536238 10.1056/NEJMoa2410819

[4] Abdul-Ghani MA, et al. Initial combination therapy with metformin, pioglitazone and exenatide is more effective than sequential add-on therapy in subjects with new-onset diabetes. Results from the Efficacy and Durability of Initial Combination Therapy for Type 2 Diabetes (EDICT): a randomized trial. Diabetes Obes Metab. 2015;17(3):268–75. 25425451 10.1111/dom.12417 PMC5577982

[5] Ludvik B, et al. Once-weekly tirzepatide versus once-daily insulin degludec as add-on to metformin with or without SGLT2 inhibitors in patients with type 2 diabetes (SURPASS-3): a randomised, open-label, parallel-group, phase 3 trial. Lancet. 2021;398(10300):583–98. 34370970 10.1016/S0140-6736(21)01443-4

[6] Nicholls SJ, et al. Cardiovascular Outcomes with Tirzepatide versus Dulaglutide in Type 2 Diabetes. N Engl J Med. 2025;393(24):2409–20. 41406444 10.1056/NEJMoa2505928

[7] UK Prospective Diabetes Study (UKPDS) Group. Intensive blood-glucose control with sulphonylureas or insulin compared with conventional treatment and risk of complications in patients with type 2 diabetes (UKPDS 33). Lancet. 1998;352(9131):837–53. 9742976

[8] Holman RR, et al. 10-year follow-up of intensive glucose control in type 2 diabetes. N Engl J Med. 2008;359(15):1577–89. 18784090 10.1056/NEJMoa0806470

[9] Group AC, et al. Intensive blood glucose control and vascular outcomes in patients with type 2 diabetes. N Engl J Med. 2008;358(24):2560–72. 18539916 10.1056/NEJMoa0802987

[10] Action to Control Cardiovascular Risk in Diabetes Study, G, et al. Effects of intensive glucose lowering in type 2 diabetes. N Engl J Med. 2008;358(24):2545–59. 18539917 10.1056/NEJMoa0802743 PMC4551392

[11] Duckworth W, et al. Glucose control and vascular complications in veterans with type 2 diabetes. N Engl J Med. 2009;360(2):129–39. 19092145 10.1056/NEJMoa0808431

[12] Ferrannini E, DeFronzo RA. Impact of glucose-lowering drugs on cardiovascular disease in type 2 diabetes. Eur Heart J. 2015;36(34):2288–96. 26063450 10.1093/eurheartj/ehv239

[13] Zinman B, et al. Empagliflozin, Cardiovascular Outcomes, and Mortality in Type 2 Diabetes. N Engl J Med. 2015;373(22):2117–28. 26378978 10.1056/NEJMoa1504720

[14] Marso SP, et al. Liraglutide and Cardiovascular Outcomes in Type 2 Diabetes. N Engl J Med. 2016;375(4):311–22. 27295427 10.1056/NEJMoa1603827 PMC4985288

[15] Gerstein HC, et al. Dulaglutide and cardiovascular outcomes in type 2 diabetes (REWIND): a double-blind, randomised placebo-controlled trial. Lancet. 2019;394(10193):121–30. 31189511 10.1016/S0140-6736(19)31149-3

[16] Bonaca MP, et al. Semaglutide and walking capacity in people with symptomatic peripheral artery disease and type 2 diabetes (STRIDE): a phase 3b, double-blind, randomised, placebo-controlled trial. Lancet. 2025;405(10489):1580–93. 40169145 10.1016/S0140-6736(25)00509-4

[17] McGuire DK, et al. Oral Semaglutide and Cardiovascular Outcomes in High-Risk Type 2 Diabetes. N Engl J Med. 2025;392(20):2001–12. 40162642 10.1056/NEJMoa2501006

[18] Marso SP, Holst AG, Vilsboll T. Semaglutide and Cardiovascular Outcomes in Patients with Type 2 Diabetes. N Engl J Med. 2017;376(9):891–2. 28249135 10.1056/NEJMc1615712

[19] Dormandy JA, et al. Secondary prevention of macrovascular events in patients with type 2 diabetes in the PROactive Study (PROspective pioglitAzone Clinical Trial In macroVascular Events): a randomised controlled trial. Lancet. 2005;366(9493):1279–89. 16214598 10.1016/S0140-6736(05)67528-9

[20] Erdmann E, et al. The effect of pioglitazone on recurrent myocardial infarction in 2,445 patients with type 2 diabetes and previous myocardial infarction: results from the PROactive (PROactive 05) Study. J Am Coll Cardiol. 2007;49(17):1772–80. 17466227 10.1016/j.jacc.2006.12.048

[21] Wilcox R, et al. Effects of pioglitazone in patients with type 2 diabetes with or without previous stroke: results from PROactive (PROspective pioglitAzone Clinical Trial In macroVascular Events 04). Stroke. 2007;38(3):865–73. 17290029 10.1161/01.STR.0000257974.06317.49

[22] Investigators OT, et al. Basal insulin and cardiovascular and other outcomes in dysglycemia. N Engl J Med. 2012;367(4):319–28. 22686416 10.1056/NEJMoa1203858

[23] Marso SP, Buse JB. Safety of Degludec versus Glargine in Type 2 Diabetes. N Engl J Med. 2017;377(20):1995–6. 29141162 10.1056/NEJMc1712575

[24] McMurray JJV, et al. Dapagliflozin in Patients with Heart Failure and Reduced Ejection Fraction. N Engl J Med. 2019;381(21):1995–2008. 31535829 10.1056/NEJMoa1911303

[25] Packer M, et al. Cardiovascular and Renal Outcomes with Empagliflozin in Heart Failure. N Engl J Med. 2020;383(15):1413–24. 32865377 10.1056/NEJMoa2022190

[26] Solomon, S.D., et al., Dapagliflozin in Heart Failure with Mildly Reduced or Preserved Ejection Fraction. N Engl J Med, 2022. 10.1056/NEJMoa2206286 36027570

[27] Kosiborod MN, et al. Semaglutide in Patients with Heart Failure with Preserved Ejection Fraction and Obesity. N Engl J Med. 2023;389(12):1069–84. 37622681 10.1056/NEJMoa2306963

[28] Packer M, et al. Tirzepatide for Heart Failure with Preserved Ejection Fraction and Obesity. N Engl J Med. 2025;392(5):427–37. 39555826 10.1056/NEJMoa2410027

[29] Heerspink HJL, et al. Dapagliflozin in Patients with Chronic Kidney Disease. N Engl J Med. 2020;383(15):1436–46. 32970396 10.1056/NEJMoa2024816

[30] Group, E.-K.C., et al., Empagliflozin in Patients with Chronic Kidney Disease. N Engl J Med, 2022.

[31] Perkovic V, et al. Effects of Semaglutide on Chronic Kidney Disease in Patients with Type 2 Diabetes. N Engl J Med. 2024;391(2):109–21. 38785209 10.1056/NEJMoa2403347

[32] Sanyal AJ, et al. Phase 3 Trial of Semaglutide in Metabolic Dysfunction-Associated Steatohepatitis. N Engl J Med. 2025;392(21):2089–99. 40305708 10.1056/NEJMoa2413258

[33] Loomba R, et al. Tirzepatide for Metabolic Dysfunction-Associated Steatohepatitis with Liver Fibrosis. N Engl J Med. 2024;391(4):299–310. 38856224 10.1056/NEJMoa2401943

[34] Lin J, et al. Effect of dapagliflozin on metabolic dysfunction-associated steatohepatitis: multicentre, double blind, randomised, placebo controlled trial. BMJ. 2025;389:e083735. 40467095 10.1136/bmj-2024-083735 PMC12135075

[35] Mandorfer M, et al. Austrian multisociety consensus on metabolic dysfunction-associated steatotic liver disease : Austrian Society of Gastroenterology and Hepatology (OGGH), Austrian Society of Diabetology (ODG), Austrian Society of Obesity (OAG). Wien Klin Wochenschr. 2025;137(Suppl 10):307–19. 41107485 10.1007/s00508-025-02617-4 PMC12534228

